# Assessment of ghrelin serum level and gene polymorphism as a risk factor in progression of chronic viral hepatitis

**DOI:** 10.1038/s41598-025-99037-1

**Published:** 2025-05-08

**Authors:** Lamiaa Mahmoud Kamel, Nagwa Mohamed Shawky, Amal A Jouda, Amany Mohamed Ibrahim, Heba H. Mahboub, Sherif Y. Mohammed

**Affiliations:** 1 Clinical Pathology Department, Faculty of Human Medicine, Zagazig University, Zagazig, Egypt; 2 Gastroenterology hepatology and infectious diseases Department, Faculty of Human Medicine, Zagazig University, Zagazig, Egypt; 3 Department of Aquatic Animal Medicine, Faculty of Veterinary Medicine, Zagazig University, Zagazig, 44511 Egypt

**Keywords:** Cirrhosis, Ghrelin, HCV, HCC, polymorphism, Biochemistry, Genetics, Molecular biology, Physiology, Diseases, Molecular medicine

## Abstract

Hepatocellular carcinoma (HCC) is a significant health concern, ranking as the fourth most common cancer in Egypt and the sixth globally. Research has identified over 300 single nucleotide polymorphisms (SNPs) in the ghrelin gene, with four of these SNPs being associated with pathogenicity. The current work is a pioneer attempt to evaluate the role of Ghrelin gene polymorphism as a risk factor for progression of chronic viral hepatitis to cirrhosis and hepatocellular carcinoma in Egyptian patients. This study was carried out on 80 cases and were allocated into four groups: Group I: apparently healthy individuals, Group II: patients with chronic viral hepatitis, Group III: patients with post-hepatitic cirrhosis, and Group IV: patients with viral hepatitis-related HCC. Serum Ghrelin was measured by ELISA Kit. Molecular detection of Ghrelin rs34911341 and rs696217 were assessed using DNA sequencing. Outcomes showed that in terms of ghrelin gene polymorphism, every group under study had a GG rs34911341. The frequency of rs696217 genotype CA was statistically significantly higher in controls than in cirrhotic and HCC cases. When cirrhosis and HCC cases were compared to controls and chronic active hepatitis cases, the serum ghrelin level decreased statistically significantly. Taken together, there was no relation of ghrelin gene polymorphism in rs34911341 with progression of chronic active hepatitis. Moreover, the frequency of rs696217 genotype CA was increased in controls compared to patients with chronic viral hepatitis and patients with viral hepatitis-related HCC. Compared to controls, liver disease patients had lower serum Ghrelin levels.

## Introduction

An estimated 8–10 million people in Egypt suffer from viral hepatitis, and millions more are at risk of contracting the illness, making it one of the country’s biggest public health issues^1^. In the globe, Egypt has the fifth-highest rate of viral hepatitis. Liver cirrhosis and cancer are more common in persons with viral hepatitis, with an expected rise in cases in Egypt in the coming years. Egypt’s leading cause of death is liver disease, which has a substantial negative impact on the nation’s health and economy^2^.

Globally, hepatocellular carcinoma (HCC) ranks third in terms of cancer-related fatalities. According to reports, HCC has an unfavorable prognosis in the end and progresses quickly. It also exhibits aggressive biological behavior. In the context of cirrhosis and chronic liver disease, HCC is developing. With a fast-rising trend, HCC ranks sixth in terms of malignancy and third in terms of cancer-related deaths^3^. The majority of HCC cases in Africa are found in Egypt, and these cases are mostly the result of chronic viral hepatitis^4^.

Ghrelin is a gastrointestinal peptide hormone that was initially discovered to be a distinct ligand for the growth hormone secretagogue receptor in the stomachs of rats and humans. In addition to stimulating stomach acid secretion and controlling gastrointestinal motility, ghrelin also regulates energy homeostasis^5^. Ghrelin’s hepatoprotective properties against liver damage and its ability to mitigate the fibrogenic response in chronically injured tissues suggest its potential as a therapeutic agent in liver diseases. Moreover, the hormone’s involvement in key cancer-related processes such as angiogenesis, apoptosis, cell migration, invasion, and proliferation highlight its potential role in tumor biology and cancer progression^6^.

The ghrelin gene comprises four introns and five exons and is found on chromosome 3p25–26^7^. According to the SNP database, there are a minimum of 263 single nucleotide polymorphisms (SNPs) in ghrelin^8^. The Ghrelin gene has four SNPs that have been thoroughly investigated: rs26311, rs27647, rs696217, and rs34911341. Furthermore, the expression and functionality of the Ghrelin protein may be impacted by these genetic changes in the Ghrelin gene, which could modify the risk of disease^9^. The objective of this study is to evaluate the influence of polymorphisms in the Ghrelin gene on the progression of chronic viral hepatitis to cirrhosis and hepatocellular carcinoma in Egyptian patients. Specifically, we aim to investigate whether certain genetic variations in the Ghrelin gene are associated with an increased risk of disease progression in these patients. By analyzing the genetic profiles of individuals with chronic viral hepatitis, we hope to identify potential biomarkers that could help in predicting disease outcomes.

## Subjects and methods

### Study design

This study was carried out in Zagazig University’s Faculty of Medicine’s Clinical Pathology and Tropical Medicine departments, used a case-control approach. This study was done between April 2022 and August 2024. Every patient read and signed the study consent form before being enrolled.

### Subjects

The participants were categorized into four groups: Group I included 20 individuals who seemed to be in good health, Group II included 20 patients with chronic viral hepatitis, Group III consisted of 20 patients diagnosed with post-hepatitic cirrhosis, and Group IV comprised 20 patients with hepatocellular carcinoma associated with viral hepatitis.

This study comprised Egyptian individuals with chronic viral hepatitis B or C (diagnosed by positive viral markers and PCR), post-hepatitic cirrhosis (diagnosed by a combination of clinical, radiographic, and laboratory evidence), and viral hepatitis-related HCC (confirmed by triphasic CT). They will be chosen at random from among the patients that attend Zagazig University Hospital.

Patients under the age of 18 and those who refused to provide informed consent for participation in the trial were excluded. Patients with other types of liver disease, such as cardiac cirrhosis, excessive alcohol consumption, drug-induced liver disease, non-alcoholic steatohepatitis, autoimmune hepatitis, metabolic, and congenital liver illnesses, were also excluded.

Every patient underwent the following procedures: full History taking through interview, thorough clinical examination, abdominal ultrasonography. and laboratory investigations. Routine investigations included complete blood count, liver and kidney function tests, alpha fetoprotein (AFP), coagulation profile, viral markers for HCV and HBV. And special investigations that included Ghrelin serum level by ELISA, and ghrelin gene rs34911341 and rs696217 by DNA sequencing. Group III and IV patients were categorized using Child-Pugh score^10^ and Model for End-stage Liver Disease (MELD)score^11^. Barcelona Clinical Liver Cancer staging system (BCLC) classification schedule was performed for group IV only^12^.

### Sampling

Ten milliliters of venous blood were aseptically taken from each patient via venipuncture and divided as follows: two milliliters of the blood sample were placed in a sterile tube containing EDTA for CBC evaluation and DNA extraction. For Prothrombin time (PT) analysis, two milliliters were put into a vacutainer tube with trisodium citrate, and six milliliters were delivered into three sterile plain vacutainer tubes with stoppers (each with two milliliters), which were left to coagulate at 37 °C for 10 min before being centrifuged for 10 min at 1200 xg. Serum from one tube was then utilized to test liver and kidney function, as well as AFP. Serum from the second tube was utilized to detect viral markers and the third tube was utilized for polymerase chain reaction (PCR) detection of HCV and HBV.

## Methods

### Routine laboratory tests

The automated cell counter, model XS 500i (Sysmex, Japan), was used to perform the complete blood count (CBC). The automated CA1500 blood coagulation analyzer (Sysmex, Japan) was used to evaluate the PT. Liver and kidney functions tests were done by German company Roche Diagnostics’ Cobas 8000 C702 module. Viral markers and AFP were evaluated by Cobas 8000 e602 module (Roche diagnostics, Germany). AmpliPrep/COBAS^®^ TaqMan PCR analyzer (Roche diagnostics, Germany) was utilized to quantify viral loads.

### Measurement of Ghrelin serum level

Ghrelin was measured in serum samples by Human GHRL(Ghrelin) ELISA Kit (Fine Biotech Co., Ltd., China) (Catalogue number: EH0355). This kit was based on the competitive-ELISA.

### Molecular detection of Ghrelin rs34911341 and rs696217 mutations

Both SNPs were assessed by DNA sequencing. The white blood cells’ DNA was extracted out using DNA blood minikit (QIAGEN-Germany). Then, amplification of the extracted DNA by PCR using specific primers. Primers sequences were as follow: forward; 5′ GCTGGGCTCCTACCTGAGC-3′, and reverse; 5’GGACCCTGTTCACTGCCAC-3′. Primers were purchased as lyophilized agents (Synbio, USA). Twelve and a half µL of one-step PCR mixture from Bio-Basic Inc. (Ontario, Canada), 2 µL of DNA template, 1 µL of each primer, and 8.5 µl of nuclease-free water were used in a 25 µl volume for the PCRs. The Gene Amp, PCR system 2400 (Perkin Elmer, AB applied biosystem, USA) thermocycler was used to do PCR, 35 cycles of 95 °C for 30 s, 60 °C for 1 min, and 72 °C for 1 min are performed after a first denaturation step at 95 °C for 1 min. The last extension was then carried out for 10 min at 72 °C. Product was detected on 2% agarose gels using ethidium bromide staining beneath a UV transilluminator. The amplified PCR products were seen at the band 618 bp. Primary purification for amplified gene by quick PCR Purification Kit (QIAGEN-Germany). After purification of PCR product, the concentration of PCR product was measured by Qubit 3.0 Fluorometer (Invitrogen, life technology, Malaysia).

Applied Biosystem’s V3.1 big dye terminator ready reaction cycle sequencing kit was used for the cycle sequencing. Secondary purification for the cycle sequencing product by US-made Applied Biosystem big dye x terminator purification kit. The Ghrelin gene mutation identification using Applied Biosystems DNA sequencers (AB 3500 genetic analyzer). In order to identify regions of similarity between sequences, the Basic Local Alignment Search Tool (BLAST) was used to analyze the sequencing results.

### Statistical analysis

The distribution pattern of the research parameters was examined using the Shapiro-Wilk test. The Kruskal-Wallis H test and posthoc test (Dunn’s test) or one-way ANOVA and posthoc test (LSD) were employed to compare several parameters. When relevant, the Chi-squared test was used. The cutoff value was determined through the use of receiver operating characteristic (ROC) analysis. The correlation coefficient method was utilized to determine the association between the examined indicators and illness characteristics. The statistical program SPSS 20.0 (Chicago, IL, USA) was employed in this investigation, with a p-value < 0.05 indicating significance.

## Results

The laboratory, clinical, and demographic data of the participants are displayed in Tables 1 and 2. The frequency of splenomegaly and MELD score increased statistically significantly among Group III compared to Group IV. Also, there was a statistically significant increase in frequency of child A among Group IV compared to Group III. According to BCLC classification, 20% of the cases were stage D and 10% were intermediate. Table 2 shows that every liver function test showed a statistically significant difference between the groups under study.

**Table 1:** Demographic and clinical characteristics of the studied groups.

Variable	Group I (*n* = 20)	Group II (*n* = 20)	Group III (*n* = 20)	Group IV (*n* = 20)	*p*
Age (years)	56.6 ± 4.76	66.4 ± 5.05	67.05 ± 4.02	65.8 ± 6.44	0.21
Sex Male/ Female	16/4 (80/20)	11/9 (55/45)	18/2 (90/10)	13/7 (65/35%)	0.06
HCV Abs	0 (0)	18 (90)	20 (100)	20 (100)	< 0.001*
HBVs Ag	0 (0)	6 (30)	4 (20)	0 (0)	0.04*
PCR HCV	0 (0)	18 (90)	0 (0)	0 (0)	< 0.001*
PCR HBV	0 (0)	6 (30)	4 (20)	0 (0)	0.04*
Hepatomegaly	–	–	0 (0)	0 (0)	1
Splenomegaly	–	–	15 (75)	0 (0)	< 0.001*
Ascites:	–	–	5 (25)	4 (20)	0.71
Coma:	–	–	2 (10)	2 (10)	1
Child-Pugh score:					
A	–	–	4 (20)	12 (60)	0.03*
B	–	–	11 (55)	4 (20)	
C	–	–	5 (25)	4 (20)	
MELD	–	–	18.5 [7–27]	9 [8–23]	0.02*
BCLC					
Very early	–	–	–	6 (30)	
Early	–	–	–	8 (40)	–
Intermediate	–	–	–	2 (10)	
Stage D	–	–	–	4 (20)	
Tumor size (mm)	–	–	–	12.44 [4.8–130]	–

Data are presented as No. (%) or median [range] or mean ± SD.

HCV: Hepatitis C virus; HBV: Hepatitis B virus; PCR: Polymerase chain reaction; MELD: Model for end-stage liver disease; BCLC: Barcelona clinic liver cancer.

* Significant.

**Table 2:** Laboratory characteristics of the studied patients.

Variable	Group II (*n* = 20)	Group III (*n* = 20)	Group IV (*n* = 20)	*p*	Post hoc
TLC (x10^3^/mm^3^)	6.45 [4–13]	4.85 [2.4–12.2]	5.5 [2.5–29.8]	0.12	–
Hemoglobin(g/dL)	13.19 ± 0.80	9.86 ± 1.12	10.31 ± 2.24	< 0.001*	< 0.001^*1^ < 0.001^*2^ 0.61 ^3^
Platelets(x10^3^/mm^3^)	180 [150–250]	77.5 [39–428]	171.5 [108–230]	0.002*	0.002^*1^ 0.07 ^2^ 0.17 ^3^
PT (Sec)	11.49 ± 0.81	15.6 ± 3.63	18.41 ± 6.95	0.003*	0.09 ^1^ 0.002^*2^ 0.33 ^3^
PTT(Sec)	34.2 ± 1.99	37.1 ± 3.48	43.25 ± 17.83	0.03*	0.66 ^1^ 0.02^*2^ 0.17 ^3^
INR	0.98 ± 0.06	1.32 ± 0.32	1.56 ± 0.91	0.008*	0.16 ^1^ 0.005^*2^ 0.35 ^3^
BUN (mg/dL)	12 [6.9–25]	26.5 [6.8–133]	25.5 [7.9–97]	0.06	–
Creatinine (mg/dL)	0.95 [0.8–1.1]	0.88 [0.46–2.6]	0.97 [0.6–7.2]	0.73	–
Total bilirubin (mg/dL)	0.6 [0.4–1.1]	2[ 0.7–7.5]	0.95 [0.3–25.5]	< 0.001*	< 0.001^*1^ 0.006^*2^ 0.15 ^3^
Direct bilirubin (mg/dL)	0.2 [0.1–0.3]	1.06 [0.3–5.6]	0.3 0.12–23.2	< 0.001*	< 0.001^*1^ 0.01^*2^ 0.01*^3^
ALT (U/L)	56 [12–133]	23 [12–47]	24.5 [13–413]	0.02*	0.001^*1^ 0.12 ^2^ 0.82 ^3^
AST (U/L)	75 [14–145]	41 [22–68]	27.5 [16–901]	0.02*	0.02^*1^ 0.04^*2^ 0.06 ^3^
Total protein (g/dL)	7.05 ± 0.30	6.16 ± 0.53	6.44 ± 0.49	< 0.001*	< 0.001^*1^ < 0.001^*2^ 0.14 ^3^
Albumin(g/dL)	4.06 ± 0.17	2.6 ± 0.23	3.37 ± 0.75	< 0.001*	< 0.001^*1^ < 0.001^*2^ < 0.001^*3^
AFP (ng/ml)	5[1–10]	16.6[6–40]	71.5[6-530]	< 0.001*	0.001^*1^ < 0.001^*2^ 0.04^*3^

Data are presented as median [range] or mean ± SD.

*TLC* total leucocyte count, *PT*, prothrombin time, *PTT* partial thromboplastin time, *INR* international normalizing ratio, *BUN* blood urea nitrogen, *ALT* alanine aminotransferase, *AST* aspartateaminotransferase.

* Significant.

P^1^: Group II versus III; P^2^: Group II versus IV; P^3^: Group III versus IV.

Regarding Ghrelin gene polymorphism among the studied groups, all studied groups were GG in rs34911341. Group I had statistical significance increase in frequency of rs696217genotype CA compared to Group II & IV (Table 3).

**Table 3:** Ghrelin gene polymorphism among the studied groups.

SNPs		Group I (*n* = 20)		Group II (*n* = 20)		Group III (*n* = 20)		Group IV (*n* = 20)		*p*
		*N*	%	*N*	%	*N*	%	*N*	%	
rs34911341	*GG*	20	100	20	100	20	100	20	100	–
rs696217	*CC* *CA*	14 6	70 30	20 0	100 0	18 2	90 10	20 0	100 0	0.004*

*Significant.

**Fig. 1:**
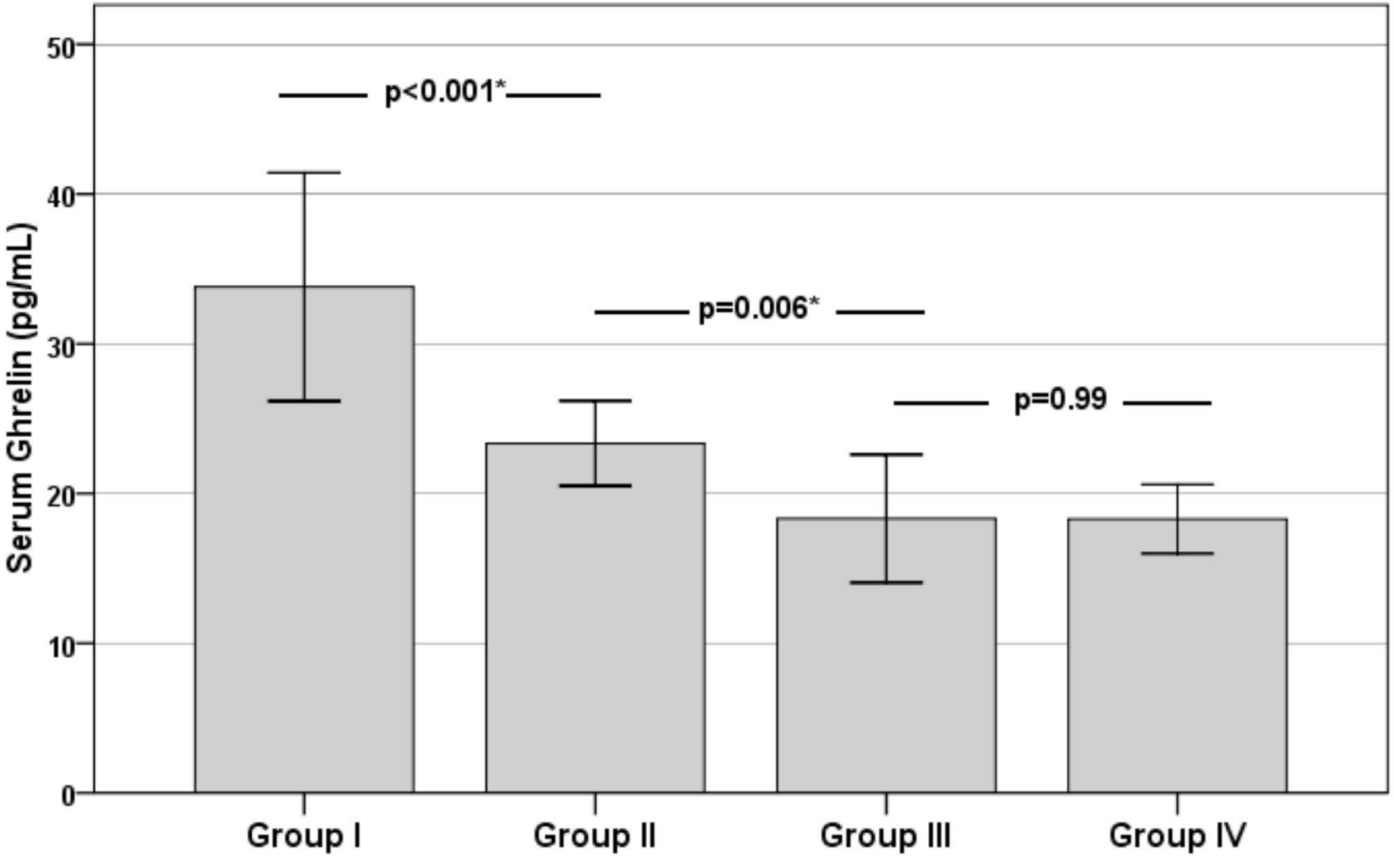
Bar plot illustrating the serum Ghrelin levels across different subject groups. *Significant.

Serum Ghrelin levels in the groups under study differed statistically significantly, as seen in Fig. 1. The Post hoc test revealed that Group III & IV showed statistical significance decrease in Ghrelin level compared to Group I& II. Also, Group II had a statistical decrease in Ghrelin compared to Group I.

Table 4 demonstrates that among the examined case groups, there was a statistically significant negative association between Ghrelin and tumor size, PT, PTT, INR, total, and direct bilirubin. Also, there was a statistically significant positive correlation between Ghrelin and total protein and albumin among the studied cases. There was no relation between clinical data and Ghrelin level in Group III. However, compared to other cases, there was a statistically significant drop in Ghrelin levels in Group IV among cases with ascites, coma, and child score C. There was a statistically significant decrease in Ghrelin level among cases with stage D in BCLC classification compared to other cases.

**Table 4:** Correlation between serum Ghrelin and different parameters among the studied cases.

Variable	Serum Ghrelin	
	Total cases (*n* = 60)	
	R	P
Age	0.01	0.96
MELD score	-0.11	0.50
Tumor size	-0.48	0.03*
TLC	-0.03	0.98
Hemoglobin	0.14	0.30
Platelets	0.24	0.07
PT	-0.58	< 0.001*
PTT	-0.31	0.02*
INR	-0.59	< 0.001*
BUN	0.08	0.54
Creatinine	-0.23	0.08
Total bilirubin	-0.49	< 0.001*
Direct bilirubin	-0.46	< 0.001*
ALT	0.18	0.17
AST	0.22	0.09
Total protein	0.58	< 0.001*
Albumin	0.35	0.007*

*TLC* total leucocyte count, *PT* prothrombin time, *PTT* partial thromboplastin time, *INR* international normalizing ratio; *BUN* blood urea nitrogen, *ALT* alanine aminotransferase, *AST* aspartate aminotransferase.

* Significant.

**Fig. 2:**
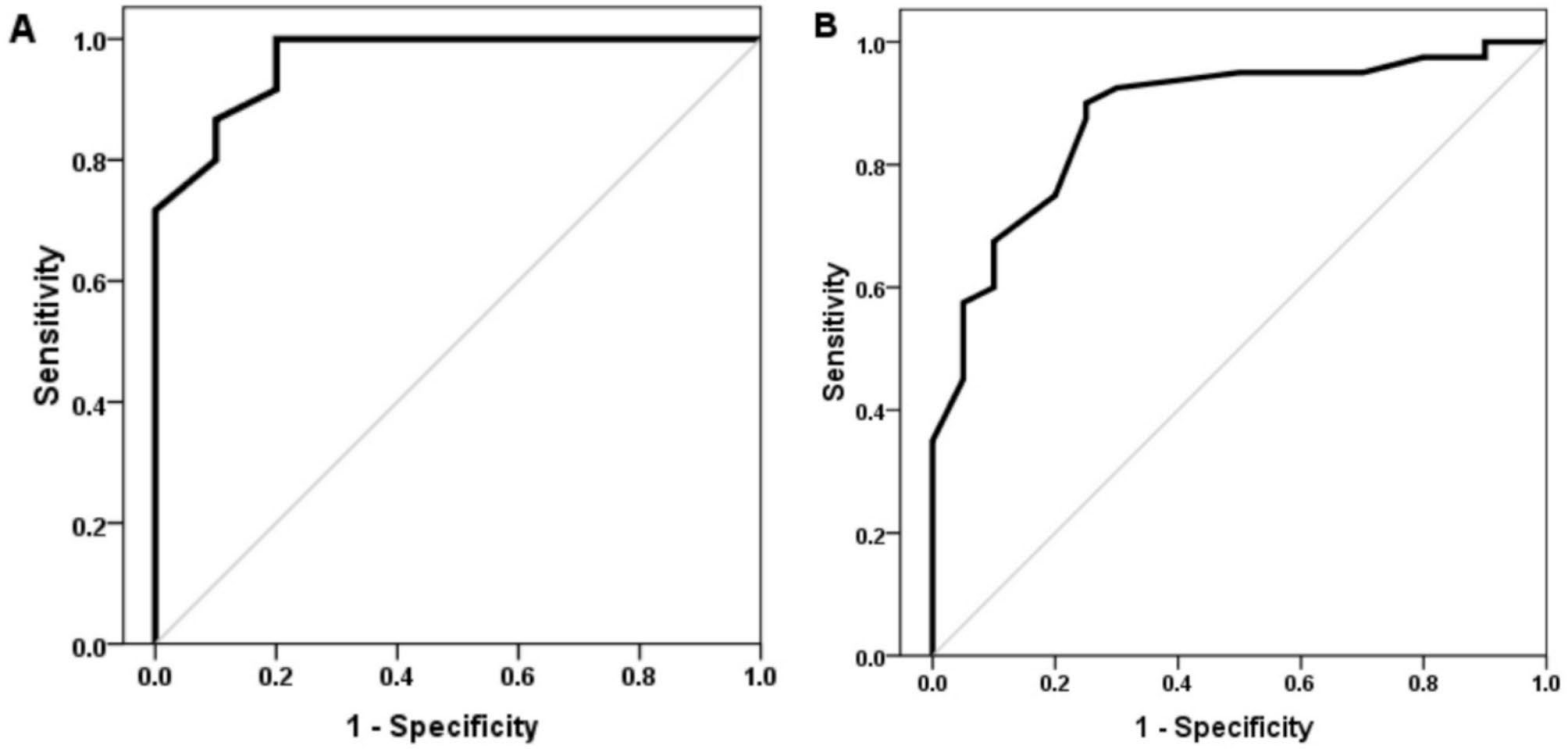
Receiver Operating Characteristic (ROC) Curve Analysis for Serum Ghrelin Levels (**A**) ROC curve illustrating the differentiation between healthy controls and patients, (**B**) ROC curve demonstrating the ability of serum ghrelin levels to differentiate between patients with cirrhosis and those without cirrhosis.

Furthermore, an examination of the ROC curve was done. The serum Ghrelin areas under the ROC curves (AUC) in differentiation of healthy controls from all cases was 0.97 (95% CI: 0.93-1) (Fig. 2A). The AUC of serum Ghrelin in differentiation between cases with cirrhosis and cases without 0.88 (95% CI: 0.79–0.97) (Fig. 2B). Serum Ghrelin at cut off < 28.5 pg/mL had sensitivity 98.3%, specificity 94.1% and accuracy 93.8% in differentiation between cases and healthy control. Serum Ghrelin at cut off < 21.9pg/ml had sensitivity 90%, specificity 75% and accuracy 85% in differentiation between cases with and without cirrhosis.

## Discussion

Liver disease represents a significant health challenge in Egypt, contributing to high mortality rates and an extensive medical and economic burden^2^. Although the prevalence of chronic viral hepatitis has declined following a nationwide control initiative between 2014 and 2018, it continues to pose a serious public health threat, with Egypt accounting for the majority of HCC cases in Africa^13,14^. Our study focuses on examining the role of polymorphisms in the ghrelin gene, where at least 300 single nucleotide polymorphisms (SNPs) exist, four of which have been implicated in potential pathogenic effects^9^. We aim to evaluate how these ghrelin gene variations contribute to the development of cirrhosis and HCC among Egyptians with chronic viral hepatitis.

The current study revealed a higher range of MELD score and a higher Child score in the cirrhotic group than in the HCC group, Moreover, the cirrhotic group had more patients with splenomegaly and a greater degree of ascites than the HCC group, that may be related to the prevalence of patients in the HCC group without liver cirrhosis; Deasai et al.^15^ stated that 20% of HCC can occur without liver cirrhosis. Different adipokines affect hepatic function as presented by Buechler et al.^16^ In contrast to our findings, Ataseven et al.^17^ studied ghrelin and leptin levels in cirrhosis and HCC driven on by the hepatitis B and D viruses and found that the groups with liver cirrhosis and those with HCC did not differ in terms of Child score. The majority of the individuals in their study had early-stage cancer, which could be the cause of this.

Liver enzymes were significantly higher in group II than group III and group IV, Zhang et al.^8^ agreed with our results, as they found that patients with chronic viral hepatitis had considerably higher levels of ALT and AST than those with liver cirrhosis and HCC. Also, chronic viral hepatitis patients with high ALT levels have an increased risk of HCC^18^.

As regard to the serum Ghrelin level among studied groups, we found that cirrhotic and HCC showed statistically significant decrease in serum Gherlin level compared to Group I and II. Our results were consistent with Elaghori et al.^19^ whose study showed that when they examined the plasma ghrelin levels of patients with liver cirrhosis (both compensated and decompensated), they discovered that the levels of cirrhosis patients were lower than those of normal control participants. Our findings supported those of Zhang et al.^8^, who revealed that, in comparison to the control group, the blood ghrelin level was significantly less in patients with HCC and liver cirrhosis. Moreover, when compared to control, Kawaguchi et al.^20^ showed that plasma active ghrelin was reduced in liver disease associated with HCV and HBV. However, they reported that in both HCV and HBV-related cirrhosis, ghrelin levels were substantially reduced in relation to the severity of liver disease. However, Ataseven et al.^17^ contradicted with our results and revealed that the cirrhosis and HCC groups had significantly greater serum ghrelin levels than the control group. The lack of global standards for measuring ghrelin may be the cause of these contradictory findings, which can also vary significantly amongst assays, populations, sample sizes, and laboratories. Furthermore, ethnic variations in genetic background might also be relevant.

Regarding the Ghrelin gene polymorphism, our study revealed that all of the tested groups had the GG genotype in rs34911341. In contrast to our findings, Motawi et al.^21^ investigated the relationship between Ghrelin gene variants and HCC progress in Hepatitis C Egyptian patients. It included three groups of patients in his study, one for those with HCV without HCC, one for HCV patients with HCC, and a control group. In rs34911341, they found each of GG, GA, and AA genotypes in his studied groups, and a notable variation was seen in the total number of patients with the GG genotype among studied group.

The present perspective revealed that group I had statistically significance increase in the frequency of rs696217 genotype CA compared to Group II & IV. In discordant to our results was Zhang et al.^8^ who included in his study three groups, group with chronic viral hepatitis, group with liver cirrhosis and group with HCC, the frequency of the genotype and allele of rs696217 did not differ significantly between the groups he studied. Also, in discordant to our results was Motawi et al.^21^ who found no significant difference between the number of patients with genotype CA among his studied group. These differences may be related to his higher sample size.

Correlations between Ghrelin level and other parameters showed that there was a statistical significance negative correlation between Ghrelin and PT, PTT, INR, total & direct bilirubin among the studied cases groups. Also, there was a statistical significance positive correlation between Ghrelin and protein and albumin among the studied cases. In group IV correlation between ghrelin and CT findings showed there was a statistical significance decrease in Ghrelin level among cases with thrombosed Portal vein and cases with Stage D in BCLC classification compared to other cases, also there was significant negative correlation between ghrelin level and tumor size. There was also a statistically significant decrease in Ghrelin level among cases with ascites, coma and child score C in group IV compared to other cases. Elaghori et al.^19^ found no correlation between plasma ghrelin level and different studied laboratory parameters of cirrhotic patients except for negative correlation with serum bilirubin level. In contrast to our findings, Tacke et al.^22^ found that ghrelin plasma levels were positively associated with child classification, with high levels being associated with Child C and serious consequences such hepatic encephalopathy, ascites, and GIT hemorrhage. According to their findings, the risk of decompensation and complications rises with higher stages. Ghrelin may be able to mitigate these issues in Child C cirrhosis through a variety of metabolic processes other than GH release, such as inducing hyperglycemia, regulating energy balance, and stimulating appetite and consumption of food^23^.

Our study showed that Ghrelin at cut off < 21.9pg/ml had sensitivity 90%, specificity 75% and accuracy 85% in differentiation between cases with and without cirrhosis. Elaghori et al.^19^ ROC analysis to study plasma Ghrelin level ability for the diagnosis of cirrhosis, Ghrelin at a cutoff value less than 850 pg/mL had a sensitivity of 87.50%; specificity of 66.7%. The difference in the value of cutoff is related to the Ghrelin sample, as we estimated active Ghrelin in our study, while Elaghori et al.^19^ estimated the total Ghrelin. It is recommended to measure the active to total ghrelin ratio^24^.

Although our study found encouraging outcomes for risk factors such viral hepatitis, there are still limitations. First, we don’t have data on additional risk factors including alcohol consumption, smoking, and aflatoxin B1 exposure. Second, the current study focused only on two SNPs in the Ghrelin gene. It is advised that further SNP variations in the Ghrelin gene be identified and investigated for their connection with viral hepatitis-related illnesses. Lastly, our study’s participants were drawn from a single hospital, which might not be representative of the Egyptian population as a whole. Therefore, additional research is required for verification of the findings.

## Conclusions

Base on the study outcomes, there was no relation of ghrelin gene polymorphism in rs34911341 with progression of chronic active hepatitis. Moreover, compared to patients with viral hepatitis-related HCC and those with chronic viral hepatitis, controls had higher frequency of rs696217 genotype CA were increased in controls compared to patients with chronic viral hepatitis and patients with viral hepatitis-related HCC. Compared to controls, liver disease patients had lower serum Ghrelin levels.

## Data Availability

Data availability statementThe datasets used during the current study available from the corresponding author on reasonable request.

## References

[1] Elbahrawy, A. et al. Current situation of viral hepatitis in Egypt. *Microbiol. Immunol.* **65**(9), 352–372. 10.1111/1348-0421.12916 (2021). 33990999 10.1111/1348-0421.12916

[2] Abdelhamed, W. & El-Kassas, M. Hepatitis B virus in Egypt: the whole story. *Egypt. Liver J.* **14**, 56. 10.1186/s43066-024-00362-3 (2024).

[3] Foglia, B., Turato, C. & Cannito, S. Hepatocellular carcinoma: latest research in pathogenesis, detection and treatment. *Int. J. Mol. Sci.* **24**(15), 12224 (2023). 10.3390/ijms241512224 PMC10419038 37569600

[4] Siegel, R. L., Miller, K. D., Wagle, N. S. & Jemal, A. Cancer statistics, 2023. *CA Cancer J. Clin.* **73**(1), 17–48. 10.3322/caac.21763 (2023). 10.3322/caac.21763 36633525

[5] Al Musaimi, O. Exploring FDA-Approved Frontiers: Insights into natural and engineered peptide analogues in the GLP-1, GIP, GHRH, CCK, ACTH, and α-MSH realms. *Biomolecules* **14**(3), 264. 10.3390/biom14030264 (2024). 38540684 10.3390/biom14030264 PMC10968328

[6] Kasprzak, A. & Adamek, A. Role of the Ghrelin system in colitis and hepatitis as risk factors for Inflammatory-Related cancers. *Int. J. Mol. Sci.* **23**(19), 11188. 10.3390/ijms231911188 (2022). 36232490 10.3390/ijms231911188 PMC9569806

[7] Al-Nbaheen, M. S. Relationship between single nucleotide polymorphism studies in Ghrelin gene with obesity subjects. *J. King Saud Univ. - Sci.* **35**(1), 102393. 10.1016/j.jksus.2022.102393 (2023).

[8] Zhang, X., Zhai, L., Rong, C., Qin, X. & Li, S. Association of Ghrelin gene polymorphisms and serum Ghrelin levels with the risk of hepatitis B Virus-Related liver diseases in a Chinese population. *PLoS One*. **10**(11), e0143069. https://doi.org/10.1371%2Fjournal.pone.0143069 (2015). 26599409 10.1371/journal.pone.0143069 PMC4658098

[9] Prodan, A. et al. Effect of the GHRL gene (rs696217) polymorphism on the metabolic disorders in patients with obesity in the Ukrainian population. *Endocr. Regul.* **57**(1), 173–182. 10.2478/enr-2023-0021 (2023). 37715984 10.2478/enr-2023-0021

[10] Johnson, P. J., Pinato, D. J., Kalyuzhnyy, A. & Toyoda, H. Breaking the Child-Pugh dogma in hepatocellular carcinoma. *J. Clin. Oncol.* **40**(19), 2078–2082. 10.1200/jco.21.02373 (2022). 35344390 10.1200/JCO.21.02373

[11] Jamil, Z. et al. Score and Glasgow Blatchford score to predict the In-Hospital outcome of portal hypertensive patients presenting with upper Gastrointestinal bleeding: an experience from tertiary healthcare system. *J. Clin. Med.* **11**(22), 6654. 10.3390/jcm11226654 (2022). 36431131 10.3390/jcm11226654 PMC9693334

[12] Morgan, M. et al. Liver cancer (BCLC staging). Reference article, Radiopaedia.org. 10.53347/rID-34362(Accessed on 15 Nov 2024) .

[13] Kandeel, A. et al. Evidence for the elimination of viral hepatitis B and C in Egypt: results of a nationwide survey in 2022. *Liver Int.* **44**(4), 955–965. 10.1111/liv.15843 (2024). 38291807 10.1111/liv.15843

[14] Jaber, F., Cholankeril, G. & El-Serag, H. B. Contemporary epidemiology of hepatocellular carcinoma: Understanding risk factors and surveillance strategies. *J. Can. Association Gastroenterol.* **7**(5), 331–345. 10.1093/jcag/gwae025 (2024). 10.1093/jcag/gwae025 PMC11477987 40786815

[15] Desai, A., Sandhu, S., Lai, J. P. & Sandhu, D. S. Hepatocellular carcinoma in non-cirrhotic liver: A comprehensive review. *World J. Hepatol.* **11**(1), 1–18. 10.4254/wjh.v11.i1.1 (2019). 30705715 10.4254/wjh.v11.i1.1 PMC6354117

[16] Buechler, C., Haberl, E. M., Rein-Fischboeck, L. & Aslanidis, C. Adipokines in liver cirrhosis. *Int. J. Mol. Sci.* **18**(7), 1392. 10.3390/ijms18071392 (2017). 28661458 10.3390/ijms18071392 PMC5535885

[17] Ataseven, H. et al. The levels of Ghrelin, leptin, TNF-alpha, and IL-6 in liver cirrhosis and hepatocellular carcinoma due to HBV and HDV infection. *Mediators Inflamm.* **2006**(4), 78380. 10.1155/mi/2006/78380 (2006). 17047295 10.1155/MI/2006/78380 PMC1618941

[18] Chen, Y. C. et al. High-normal and abnormal Alanine transaminase levels linked to increased risk of hepatoma following treatment for chronic hepatitis C. *J. Formos. Med. Assoc.*10.1016/j.jfma.2025.01.026 (2025). 10.1016/j.jfma.2025.01.026 39919992

[19] Elaghori, A., Salem, P. E. S., Azzam, E. & Abu Elfotoh, N. Ghrelin level in patients with liver cirrhosis. *Acta Endocrinol. (Buchar)*. **5**(1), 62–68 (2019). 10.4183/aeb.2019.62 PMC6535318 31149061

[20] Kawaguchi, T., Nagao, Y. & Sata, M. Independent factors associated with altered plasma active Ghrelin levels in HCV-infected patients. *Liver Int.* **33**(10), 1510–1516. 10.1111/liv.12235 (2013). 23809581 10.1111/liv.12235

[21] Motawi, T. K., Shaker, O. G., Ismail, M. F. & Sayed, N. H. Genetic variants associated with the progression of hepatocellular carcinoma in hepatitis C Egyptian patients. *Gene* **527**(2), 516–520. 10.1016/j.gene.2013.06.053 (2013). 23845776 10.1016/j.gene.2013.06.053

[22] Tacke, F. et al. Ghrelin in chronic liver disease. *J. Hepatol.* **38**(4), 447–454. 10.1016/s0168-8278(02)00438-5 (2003). 12663236 10.1016/s0168-8278(02)00438-5

[23] El-Shehaby, A. M., Obaia, E. M., Alwakil, S. S. & Hiekal, A. A. Total and acylated Ghrelin in liver cirrhosis: correlation with clinical and nutritional status. *Scand. J. Clin. Lab. Invest.* **70**(4), 252–258. 10.3109/00365511003763349 (2010). 20367557 10.3109/00365511003763349

[24] Garcia, J. M. et al. Active Ghrelin levels and active to total Ghrelin ratio in cancer-induced cachexia. *J. Clin. Endocrinol. Metab.* **90**(5), 2920–2926. 10.1210/jc.2004-1788 (2005). 15713718 10.1210/jc.2004-1788

